# Roles of TRAFs in Ischemia-Reperfusion Injury

**DOI:** 10.3389/fcell.2020.586487

**Published:** 2020-11-05

**Authors:** Wei Zhou, Danni Lin, Zibiao Zhong, Qifa Ye

**Affiliations:** ^1^Zhongnan Hospital of Wuhan University, Institute of Hepatobiliary Diseases of Wuhan University, Transplant Center of Wuhan University, Hubei Key Laboratory of Medical Technology on Transplantation, Engineering Research Center of Natural Polymer-based Medical Materials in Hubei Province, Wuhan, China; ^2^The First Affiliated Hospital, Zhejiang University School of Medicine, Department of Hepatobiliary and Pancreatic Surgery, Zhejiang Provincial Key Laboratory of Pancreatic Disease, Innovation Center for the Study of Pancreatic Diseases, Hangzhou, China; ^3^The Third Xiangya Hospital of Central South University, Research Center of National Health Ministry on Transplantation Medicine Engineering and Technology, Changsha, China

**Keywords:** TRAFs, ischemia-reperfusion injury, hypoxia/reoxygenation, inflammation, ubiquitination

## Abstract

Tumor necrosis factor receptor-associated factor (TRAF) proteins are a family of signaling molecules that function downstream of multiple receptor signaling pathways, and they play a pivotal role in the regulation of intracellular biological progresses. These TRAF-dependent signaling pathways and physiological functions have been involved in the occurrence and progression of ischemia-reperfusion injury (IRI), which is a common pathophysiological process that occurs in a wide variety of clinical events, including ischemic shock, organ transplantation, and thrombolytic therapy, resulting in a poor prognosis and high mortality. IRI occurs in multiple organs, including liver, kidney, heart, lung, brain, intestine, and retina. In recent years, mounting compelling evidence has confirmed that the genetic alterations of TRAFs can cause subversive phenotype changes during IRI of those organs. In this review, based on current knowledge, we summarized and analyzed the regulatory effect of TRAFs on the IRI of various organs, providing clear direction and a firm theoretical basis for the development of treatment strategies to manipulate TRAF proteins or TRAF-dependent signaling pathways in IRI-related diseases.

## Background

**FIGURE 1:**
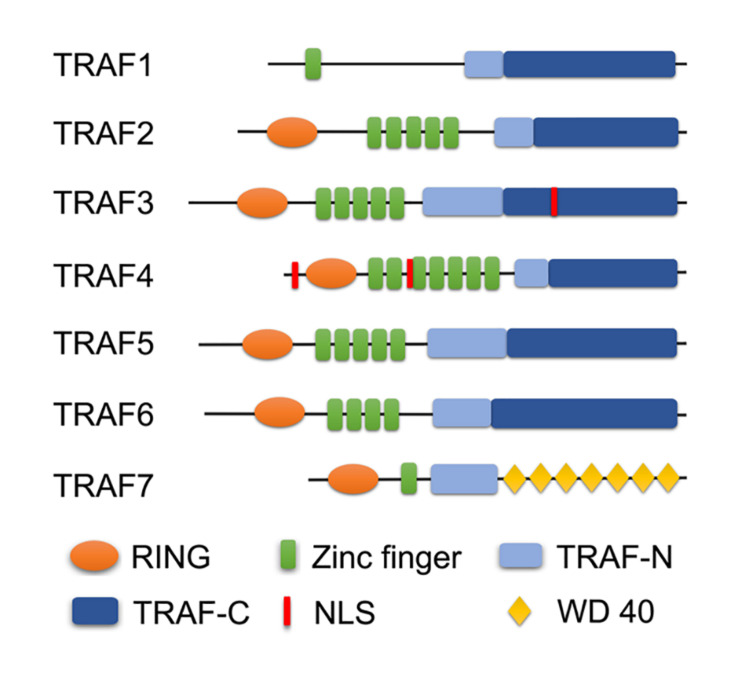
Domain organization of TRAF family. A schematic diagram represents the basic structural features of TRAF1-7. All TRAF members, except TRAF1, contain a similar N-terminal RING domain, followed by one or more zinc fingers. Most TRAFs share a similar TRAF domain divided into an N-terminal coiled-coil domain (TRAF-N) and a C-terminal β-sandwich domain (TRAF-C or MATH domain), except TRAF7 (in which seven WD40 repeats replace the TRAF domain). Especially, TRAF3 and TRAF4 contain the nuclear localization signal (NLS) sequence.

Tumor necrosis factor receptor-associated factors (TRAFs) were identified as the signaling adaptors that positively and negatively regulate the signal transduction pathways of various receptors, including the TNF-R superfamily, Toll-like receptors (TLRs), NOD-like receptors (NLRs), RIG-I-like receptors (RLRs), and cytokine receptors (Fang et al., 2017; Dhillon et al., 2019; Swaidani et al., 2019). There are six typical members (TRAF1–6) and an atypical member (TRAF7) in mammalian cells (Park, 2018; He et al., 2020). Most TRAFs share a similar C-terminal TRAF domain, a distinct feature of the typical TRAF proteins except for TRAF7 (in which seven WD40 repeats replace the TRAF domain), which contain an N-terminal coiled-coil domain (TRAF-N) and a highly conserved C-terminal β-sandwich domain (TRAF-C or MATH domain) (Arkee and Bishop, 2019; Stevers et al., 2019). Furthermore, all TRAF members, with the exception of TRAF1, contain a similar N-terminal RING domain, followed by one or more zinc fingers (Zapata et al., 2018; Arkee and Bishop, 2019; Stevers et al., 2019; Figure 1). The TRAF domain is responsible for mediating the oligomerization between the TRAF proteins, as well as their association with upstream regulators and downstream effectors. The RING domain is found in many E3 ubiquitin ligases and is responsible for mediating proteins’ ubiquitination (Lu et al., 2018; Zapata et al., 2018; Arkee and Bishop, 2019; Stevers et al., 2019). Due to their structural characteristics, TRAF proteins are involved in a variety of intracellular pathophysiological processes, including cell apoptosis, proliferation, differentiation, autophagy, necroptosis, pyroptosis, immune, and inflammatory responses (Lan et al., 2017; Robeson et al., 2018; Dhillon et al., 2019; Li et al., 2019b; Swaidani et al., 2019; Zhang X. et al., 2019). Specially, since TRAFs were discovered in TNF-R signaling, its role has been expanded to include involvement in more and more other inflammatory cytokine receptors, such as receptors for IL-2, IL-6, IL-1β, IL-17, IL-18, IL-33, type I IFNs, type III IFNs, M-CSF, GM-CSF, and C-type lectin receptors (Dhillon et al., 2019; Swaidani et al., 2019). Certainly, as intracellular scaffolding molecules, TRAFs play an indispensable role via the complex interactions with the inflammatory cytokine receptors in regulating pathophysiological processes in many human diseases, including autoimmune diseases, cancers, atherosclerosis, and type II diabetes, and they even have been recommended as suitable targets for therapeutic intervention (Choi et al., 2018; Nagashima et al., 2018; Zhu et al., 2018; Dhillon et al., 2019; Sajjad et al., 2019; Sangare et al., 2019). Meanwhile, the biological and functional roles of TRAFs in ischemia-reperfusion injury (IRI) of various organs have received much attention in recent years.

IRI can occur in a variety of tissues and organs, including the liver, kidney, heart, lung, brain, intestine, and retina, leading to severe pathophysiological damage to the primary organ or even remote organs, partly due to explosive oxidative stress, inflammation and destruction of the physiological body barrier. The occurrence of IRI leads to a poor prognosis in many diseases, such as neuronal damage caused by ischemic shock, acute cardiopulmonary damage caused by thrombolytic therapy, and visual impairment caused by retinopathy (Chen et al., 2017; Li et al., 2019d; Hui et al., 2020; Kohler et al., 2020). However, there is still no exact treatment method in clinical guidelines due to the devastating pathological damage and complex molecular mechanisms involved in IRI. To achieve better therapeutic effects, increasing attention has been paid to the study of IRI in recent years. Previous studies have shown that the main mechanisms of IRI include apoptosis and necrosis (Zhou H. et al., 2017; Li et al., 2018a; Xu T. et al., 2019), autophagy (Russo et al., 2018; Yu et al., 2018), and activation of the complement and lymphatic systems (Gigliotti et al., 2013; Bajwa et al., 2016). The signaling pathways involved mainly include the nuclear factor-κB (NF-κB) (Li et al., 2018a; Zhang R. et al., 2018), apoptosis signal-regulating kinase 1 (ASK1) (Qin et al., 2018), transforming growth factor-β-activated kinase 1 (TAK1), and autophagy (ATG) pathways (Li et al., 2018d; Chen et al., 2020). Recently, IRI events have increasingly been shown to involve newly proposed mechanisms such as necroptosis (Linkermann et al., 2012; Li et al., 2018c; Zhou et al., 2018), pyroptosis (Qiu et al., 2017; Tajima et al., 2019), and ferroptosis (Gao et al., 2015; Tonnus and Linkermann, 2016; Li et al., 2019f), which have attracted substantial attention and keen interest.

**FIGURE 2:**
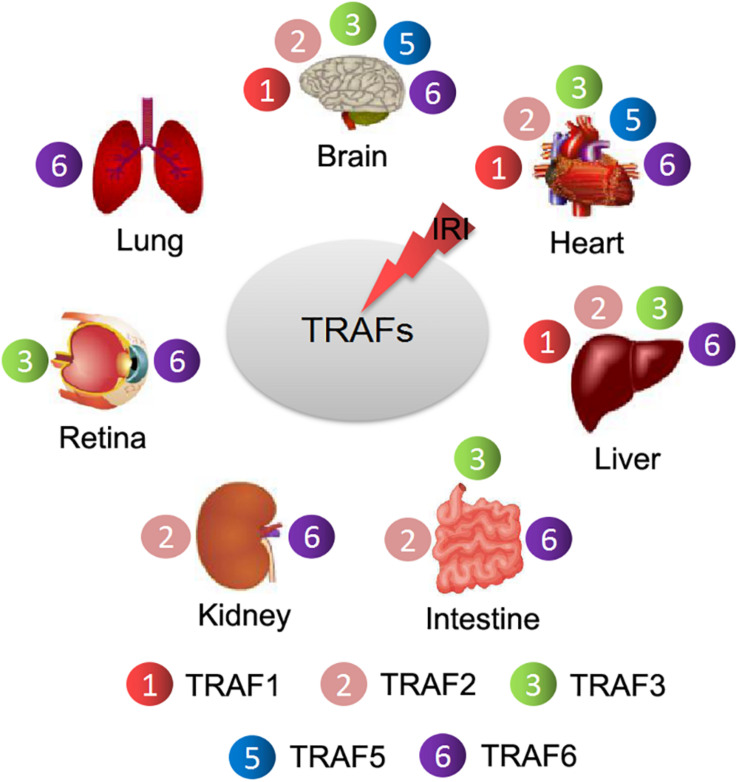
Different TRAF members are involved in the regulation of IRI in different organs. TRAF1/2/3/5/6 regulate cerebral and myocardial IRI. TRAF1/2/3/6 are involved in liver IRI. TRAF2/3/6 participate in the regulation of intestinal IRI. TRAF2/6 are related to renal IRI. TRAF3/6 are involved in retinal IRI. TRAF6 is correlated with lung IRI.

Convincing evidence in recent years has proven that most TRAF proteins, except TRAF4 and TRAF7, are involved in the development of IRI by regulating respective molecular mechanisms to play a similar or opposite effect on various organs (Figure 2). In this review, we will state and elucidate the specific role of most TRAF molecules in the IRI of different organs to provide a theoretical basis for formulating treatment strategies for diseases related to IRI, and provide directions for further TRAFs exploration.

### Basic Concept and Network Regulate of TRAFs in Cellular Signaling Cascades

#### TRAF1

TRAF1, a unique member of the TRAF family, lacks the N-terminal RING domain, whose expression is low in resting cells and is only found in activated dendrites, lymphocytes, and certain epithelia (Kim C. M. et al., 2017; Ivagnès et al., 2018). In addition, TRAF1 exerts its primary function in specific organs in mice and humans, including the spleen, lung, and testis (Wang et al., 2018). Although the structure of TRAF1 is more simple than other TRAFs and its expression is restricted, it still plays an indispensable role in regulating certain intracellular biological progresses, including cellular innate immunity and apoptosis signaling. For apoptosis, TRAF1 seems to exert conflicting biologic effects depending on the specific cell type. In immune cells, TRAF1 shows an anti-apoptotic function in TNFR2 signaling via direct interaction with TNFR2; however, it plays a pro-apoptotic function in neuronal cell death (Lu et al., 2013; Kim C. M. et al., 2017). Interestingly, TRAF1-deficient mice showed hyper-inflammation in obese visceral adipose tissue, failure to gain weight, and improved insulin resistance (Anto Michel et al., 2018). Furthermore, a study of septic, TRAF1-deficient mice showed increased susceptibility to lipopolysaccharide (LPS)-induced septic shock (Abdul-Sater et al., 2017). Clearly, based on its physiological function, TRAF1 plays an important role in many human inflammation and metabolic diseases.

#### TRAF2

TRAF2 is the most extensively studied member of the TRAF family in terms of structure and function. Similar to other TRAF proteins, TRAF2 contains the conventional N-terminal RING domain, zinc finger domain, and C-terminal TRAF domain (Bhat et al., 2018; Chen et al., 2018). Recently, a study showed that the zinc finger domain of TRAF2, instead of the conventional substrate recognition C-terminal TRAF domain, was responsible for interacting with the aryl hydrocarbon receptor nuclear translocator-like protein 1 (BMAL1) (Chen et al., 2018). Therefore, the functions of substrate recognition domains in TRAF2 differ according to the substrates.

Organizationally, TRAF2 is widely expressed in various tissues, including the brain, heart, liver, spleen, lung, kidney, testis, and skeletal muscle, and has the highest expression in the spleen in mice (Qu et al., 2017). In terms of regulation, as an E3 ubiquitin ligase, TRAF2 is regulated by complex managers, including cIAP-1, siah2 and A20 for ubiquitination and degradation (Zhang et al., 2020), and CYLD, USP4, USP25 and USP48 for the deubiquitination (Li et al., 2002, 2018b; Trompouki et al., 2003). Surprisingly, USP48 knockdown increased the abundance of lysine 48 (K48)-linked ubiquitination but not the K63-linked ubiquitination of TRAF2, and subsequently reduced its stability (Li et al., 2018b). These complex managers contribute to TRAF2’s regulation of multiple cellular responses, such as anoikis, cell proliferation, apoptosis and necroptosis, and immune and inflammatory responses (Guo et al., 2017; da Silva et al., 2019), contributing to the pathogenesis of many diseases, especially cancers, including hepatocellular carcinoma (HCC) and gastrointestinal cancers (Hirsova et al., 2017; Schneider et al., 2017; Xu et al., 2017), prostate and pancreatic cancers (Wei et al., 2017; Chen et al., 2018), lung cancer (Lu et al., 2018), and ovarian and breast cancer (da Silva et al., 2019; Zhang et al., 2020).

#### TRAF3

Anatomically, similar to most TRAF members, TRAF3 contains a conserved TRAF domain, zinc finger domain, and a RING domain (Xie et al., 2020). Interestingly, recent studies have found that the TRAF-C domain of TRAF3 contains the nuclear localization signal (NLS) sequence (Mambetsariev et al., 2016), suggesting that TRAF3 may have a biological function in the nucleus besides its canonical functions in the cell membrane and cytoplasm. However, probably due to the postnatal lethality of global TRAF3 deficiency, the functions of TRAF3 are more elusive than other members (Hu et al., 2016; Wallis and Bishop, 2018). TRAF3 functions were delayed until the mature application of gene conditional knockout (Wallis and Bishop, 2018), and primarily included a role in immune and inflammatory responses via regulating the NF-κB, mitogen-activated protein kinase (MAPK) and type I interferon (IFN-I) pathways (Perez-Chacon et al., 2018; Fochi et al., 2019). Importantly, TRAF3 plays a master role in regulating the homeostasis and function of B cells, where it is involved in their proliferation, survival, and differentiation (Perez-Chacon et al., 2018; Whillock et al., 2019). Recently, TRAF3 is getting increasing attention in various viral infections, including severe acute respiratory syndrome coronavirus (SARS-CoV) (Siu et al., 2019), hepatitis B virus (HBV) (Xie et al., 2020), human papilloma viruses (HPV) (Zhang J. et al., 2018; Xiao et al., 2019), and enterovirus 71 (EV71) (Gu et al., 2017), by regulating the IFN-I or NF-κB pathways. In SARS-CoV research, TRAF3, but not TRAF2 or TRAF6, was required for SARS-CoV open reading frame 3a (ORF3a)-induced activation of NF-κB (Siu et al., 2019). However, the effects of TRAF3 on NF-κB pathways are controversial. A prevalent view holds that TRAF3 negatively regulates the non-canonical NF-κB pathway (Li et al., 2019a,c; Zhang Z. et al., 2019), but many certified events have shown that TRAF3 can activate both canonical and non-canonical NF-κB pathways (He et al., 2004; Bista et al., 2010). NF-κB pathways are vital regulators of many pathological events, including inflammation and apoptosis, in ischemic injury (Gong et al., 2015; Xu et al., 2018). Thus, as a crucial regulator of NF-κB pathways, TRAF3 has attracted much attention in the study of IRI of various organs.

#### TRAF4

TRAF4 is a unique member of the TRAF family, in terms of both structure and function. Firstly, TRAF4 is an ancestral member, due to the fact that other TRAFs members (except TRAF6) have evolved to a certain extent (Cai et al., 2017). Secondly, TRAF4 is the only member that has three cysteine-rich motifs associated with TRAF and RING domains, followed by seven zinc fingers (Rousseau et al., 2014; Yang et al., 2015). In addition, TRAF4 has the canonical NLS sequence, similar to TRAF3 (Mambetsariev et al., 2016; Das et al., 2019). Moreover, although it has been confirmed that TRAF4 is widely expressed in adult tissues, its subcellular localization has been controversial for years (Shen et al., 2013; Yi et al., 2013). The mainstream views hold that it is broadly located in the cell membrane, cytoplasm, and nucleus (Rousseau et al., 2014; Ren et al., 2015). Perhaps due to the particularity of its structure and expression, TRAF4 plays an important role in developmental steps, such as tracheal ring formation, neural tube closure, and axial skeleton formation (Kim E. et al., 2017). However, there were no obvious immunological defects or lymphocyte changes in TRAF4-deficient mice (Kang et al., 2018), suggesting that TRAF4 may not have a significant impact on immune function in mice. Moreover, although researchers have confirmed that TRAF4 did not bind to TNF receptors, or only weakly interacted with a few TNF receptor family members under certain conditions (Ren et al., 2015), TRAF4 is a crucial regulator in the transforming growth factor-β (TGF-β), Wnt-β-catenin, and phosphatidylinositol-3-kinase (PI3K)/AKT pathways, which are involved in tumorigenesis and progression (Kang et al., 2018). However, unlike TRAF1/2/3/5/6, study about TRAF4’s involvement in IRI is few.

#### TRAF5

TRAF5 is much less studied than TRAF3, although TRAF5 shares the highest sequence with TRAF3 in the TRAF family (Foight and Keating, 2016; Kim et al., 2020). Similarly, TRAF5 contains an N-terminal RING domain, five zinc fingers at the middle of its sequence, a coiled-coil domain, and a MATH domain included in the C-terminal TRAF domain (Xu et al., 2020). TRAF5 mainly exists in immune organs, including the spleen and thymus, and is also abundantly expressed in the epidermis, lungs and kidneys (Nagashima et al., 2018; Xia et al., 2019). Based on its characteristics of expression, TRAF5 is closely related to many immune-related diseases, such as systemic lupus erythematosus (SLE), inflammatory bowel disease (IBD), and the infection of classical swine fever virus (CSFV) (Wang et al., 2015; Shang et al., 2016; Lv H. et al., 2018). In addition, TRAF5 plays a vital role in the progression of various cancers, including HCC, colorectal cancer, breast cancer, and prostate cancer, via regulating cell proliferation, apoptosis, and survival (Ling et al., 2018; Jiang et al., 2020). Interestingly, there have been studies showing that TRAF5 is associated with chronic inflammation-related diseases, which demonstrate that TRAF5 plays protective roles in atherosclerosis and obesity-induced non-alcoholic fatty liver disease or non-alcoholic steatohepatitis (Missiou et al., 2010; Gao et al., 2016).

#### TRAF6

TRAF6, one of the most evolutionarily ancient members of TRAF family, is ubiquitously expressed in various tissues and cell types (Cai et al., 2017; Jiang et al., 2017; Lalani et al., 2018). It contains a characteristic C-terminal TRAF domain, a similar N-terminal RING domain, followed by at least four zinc fingers (Hu et al., 2017; Fu et al., 2018). Similar to TRAF2/3/5, the RING domain of TRAF6 possesses non-conventional E3 ubiquitin ligase activity. Generally, K48-linked ubiquitination of TRAF6 is responsible for the degradation of substrate, whereas K63-linked ubiquitination is responsible for signaling activation and protein trafficking (Hu et al., 2017; Lv Y. et al., 2018). However, K63-linked ubiquitination can also promote the degradation of lysosomal-mediated substrate proteins (Lu et al., 2017). Interestingly, TRAF6 can be auto-ubiquitinated through K63-linked ubiquitin chains, which is a vital prerequisite for its activation (Min et al., 2018). In addition to being an E3 ubiquitin ligase, TRAF6 is also an adapter protein, which bridges between the TLRs, TNF-R superfamily, and IL-1 receptors with downstream signal pathways, especially MAPK and NF-κB pathways, regulating immune and inflammatory responses (David et al., 2018; Fu et al., 2018). Unquestionably, these receptors and pathways are fatal for cell inflammation, survival, and death (Zhou et al., 2015), which commonly occurs in the pathophysiological processes of IRI.

#### TRAF7

TRAF7, the latest identified member, is the other unique protein of the TRAF family besides TRAF4. TRAF7 is also the only atypical member in TRAF family, as it contains seven WD40 repeat domains instead of the common TRAF domain (Clark et al., 2013). Similar to the TRAF domain, the WD40 repeat domains are responsible for the protein-protein and protein-DNA interactions (Zotti et al., 2017). In addition, TRAF7 also possesses the N-terminal RING domain and the adjacent zinc finger, similar to most other TRAF members (Tokita et al., 2018). Thus far, the biological function of TRAF7 remains elusive and is under investigation. Most studies at present are about the correlativity between mutations in TRAF7 and various tumors, including meningiomas, adenomatoid tumors and intraneural perineuriomas (Reuss et al., 2013; Klein et al., 2017; Goode et al., 2018), and all such mutations are heterozygous missense mutations, which cluster within the mutational hotspots in the WD40 domains (Klein et al., 2017; Goode et al., 2018). The latest research showed that, as a E3 ubiquitin ligase like most other TRAF members, TRAF7 played an important role in the regulation of inflammatory response, cell apoptosis, and tumor progression by the lysosomal degradation of NF-κB essential modulator and the proteasomal degradation of Krüppel-like factor 4, respectively (He et al., 2020). In addition, a study showed that TRAF7 played a major role in the suppression of endothelial hyperpermeability induced by inflammatory stimuli and roundabout4, an endothelial cell-specific receptor, which could enhance the function of TRAF7 (Shirakura et al., 2019). It can be seen that TRAF7 can cooperate with other molecules to regulate inflammation and cell apoptosis. It may also play a role in IRI, but according to our knowledge, there is no relevant research in this regard now.

### Roles of TRAFs and Agents for Regulating TRAFs in IRI of Different Organs

#### TRAF1

According to current research, TRAF1 plays a vital role in cerebral, liver, and myocardial IRI (Lu et al., 2013; Zhang et al., 2014; Huang X. et al., 2019; Xu W. et al., 2019). Cerebral IRI is the main culprit causing ischemic stroke, accounting for ∼80% of total stroke cases, which has caused adult neurological disability globally and presented a very high mortality rate yearly (Li et al., 2019d,e). Current treatment strategies are limited for ischemic stroke because the mechanisms of cerebral IRI are extremely complicated. Surprisingly, TRAF1 transgenic (TG-TRAF1) mice showed enlarged stroke lesions while TRAF1-deficient (TRAF1-KO) mice showed significant lesion reduction (Lu et al., 2013). Meanwhile, an *in vitro* experiment also showed that increased TRAF1 expression resulting from the infection of adenovirus-harboring human TRAF1 cDNA (Ad-TRAF1) presented more neuronal apoptosis than decreased TRAF1 expression caused by treatment with TRAF1 short hairpin RNA (Ad-shTRAF1) (Lu et al., 2013). Mechanistically, TRAF1 promoted ischemic cerebral injury by directly interacting with ASK1 (Lu et al., 2013). Furthermore, it may be the N-terminal region or kinase domain of ASK1, rather than the C-terminal region, that is capable of interacting with TRAF1, and that the TRAF domain is essential for TRAF1-induced neuronal injury after ischemia. Moreover, Fas-/FasL-regulated necroptosis was also involved in the process of TRAF1 cerebral IRI regulation (Lu et al., 2013). Unfortunately, there was no in-depth exploration of the relationship between TRAF1 and necroptosis in the study.

Subsequently, it has also been verified that the TRAF1/ASK1 axis promotes liver IRI and myocardial IRI (Zhang et al., 2014; Huang X. et al., 2019; Xu W. et al., 2019). Liver IRI is a common pathological process that occurs in hemorrhagic shock, trauma, liver resection, and liver transplantation, which lead to early allograft dysfunction (EAD), an important cause of morbidity and mortality in liver transplant recipients (Monga, 2018; Ni et al., 2019; Nakamura et al., 2020). TRAF1 deficiency inhibited inflammation and cell death, and TRAF1-KO mice were resistant to liver IRI (Zhang et al., 2014). *In vitro*, the overexpression of miR-214 reduced hepatocyte apoptosis following hypoxia/reoxygenation (H/R) injury by negatively regulating the TRAF1/ASK1/JNK pathway (Huang X. et al., 2019). In myocardial IRI, TRAF1-KO mice showed decreased cardiomyocyte apoptosis and milder inflammatory response than the TRAF1 wild-type (WT) mice. Explicitly, TRAF1 aggravated primary neonatal cardiomyocyte inflammation and apoptosis via promoting the ASK1/JNK/p38 cascades in response to H/R (Xu W. et al., 2019). However, TRAF1 is also expressed in fibroblasts and endothelial cells of the heart. Whether TRAF1 contributes to the regulation of non-cardiomyocytes in IRI is still unclear. Overall, current research showed that TRAF1 played an important role in cerebral, liver, and myocardial IRI, and the TRAF1/ASK1 axis was the common signaling pathway involved in IRI regulation for these organs.

#### TRAF2

Similarly, TRAF2 plays a vital role in regulating IRI of various organs, including myocardial, intestine, brain, kidney, and liver, through different molecular mechanisms (Xia et al., 2012; Tzeng et al., 2014; Tan et al., 2015; Zhou W. et al., 2017; Li et al., 2019b). Myocardial infarction remains one of the leading health problems around the world. However, recanalization of previously blocked blood vessels can cause serious myocardial IRI, which is a leading risk factor for heart failure (Zhu et al., 2019; Kohler et al., 2020). An early myocardial reperfusion period can cause endoplasmic reticulum (ER) stress and subsequently increase the expression of ER stress markers, including TRAF2 (Wang Z. H. et al., 2014). Later, a study showed that cardiac-restricted expression of dominant negative TRAF2 (MHC-TRAF2_*D*__*N*_) mice had significantly worse left ventricular (LV) functional recovery, increased Evans blue dye uptake, and increased creatine kinase (CK) release (Tzeng et al., 2014). Conversely, low levels of TRAF2 expression in the mice hearts (MHC-TRAF2_*L*__*C*_) significantly improved LV functional recovery (Burchfield et al., 2010; Tzeng et al., 2014). Clearly, TRAF2 played a cardioprotective role in myocardial IRI.

We have also proven the protective effects of TRAF2 in intestinal IRI, which occurs in a wide variety of clinical conditions, including hemorrhagic shock, acute mesenteric ischemia, and organ transplantation, resulting in a high mortality rate that range from 70 to 80% (Higuchi et al., 2008; Jia et al., 2020). In our research, we verified that PKCζ, a member of the atypical protein kinase C (aPKC) subfamily, phosphorylated TRAF2 at Ser^55^, rather than at Ser^11^, activating NF-κB but inhibiting c-Jun to attenuate cell apoptosis, leading to protection against the injury induced by intestinal ischemia-reperfusion (Zhou W. et al., 2017). In terms of cerebral IRI, the role of TRAF2 seems to be controversial in current research. One study showed that in an *in vitro* model simulating cerebral IRI, the knockdown of TRAF2 in microglia reduced neuronal injury induced by oxygen-glucose deprivation reperfusion (OGDR), and the sphingosine kinase 1 (Sphk1)/TRAF2/NF-κB pathway was responsible for the increased neuronal apoptosis following OGDR (Su et al., 2017). However, a recent study suggested that TRAF2 interacted with mixed-lineage kinase domain-like (MLKL) protein, protecting against cerebral IRI by suppressing necroptosis (Li et al., 2019b). Thus, it can be seen that TRAF2 may play different roles by interacting with different regulatory proteins in specific cell types. In renal and liver IRI, TRAF2 is only involved as a participant in these pathological processes (Ben Mkaddem et al., 2010; Kim and Lee, 2012; Xia et al., 2012; Tan et al., 2015). However, there has been no in-depth discussion of its specific mechanism. It follows that TRAF2 plays complicated role in IRI of various organs, and further research needs to be conducted.

#### TRAF3

TRAF3 is ubiquitously expressed in multiple organs, primarily including the brain, liver, heart, lung, and spleen (Liu F. et al., 2018; Dai et al., 2019). The TRAF3-related IRI was mainly concentrated in the brain, liver, heart, intestine and retina (Gong et al., 2015; Hu et al., 2016; Liu X. et al., 2018; Dai et al., 2019; Ge et al., 2020). In cerebral IRI, TRAF3 is a central regulator through its interaction with and phosphorylation of TAK1 (Gong et al., 2015). The TRAF3-TAK1 signaling pathway promoted neural cell death, inflammatory response, and oxidative stress in cerebral IRI by regulating the JNK, NF-κB, and Rac-1/NADPH oxidase pathways, respectively (Gong et al., 2015). The underlying mechanism by which TRAF3 regulates canonical NF-κB pathways remains largely unclear in previous studies. Surprisingly, the cerebral IRI research suggested that TRAF3 promoted the activation of canonical NF-κB pathways via phosphorylation of TAK1 (Gong et al., 2015), which provided new evidence for TRAF3’s role in regulating the canonical NF-κB pathway. Similarly, TRAF3 promoted the liver damage and inflammation induced by liver IRI by directly binding to TAK1, which activated the downstream JNK and NF-κB pathways (Hu et al., 2016). The effects of TRAF3 on JNK pathways were focused on myocardial IRI, which showed that TRAF3 promoted apoptosis, inflammation, and oxidative stress in the hearts of mice with IRI by JNK activation (Liu X. et al., 2018). Although TRAF3 initially exerted a negative regulatory effect on the JNK pathway (Matsuzawa et al., 2008), these IRI studies have shown that TRAF3 can activate JNK under certain conditions.

Additionally, the effects of TRAF3 on NF-κB pathways were verified in intestinal IRI. TRAF3’s regulation of NF-κB is time-dependent in intestinal IRI. Research showed that TRAF3 promoted the expression of NF-κB at 90 min of reperfusion *in vivo*; however, it inhibited the expression of NF-κB with further prolongation, and the results were verified *in vitro* (Dai et al., 2019). Moreover, research identified TRAF3 as a target gene of miR-29b-3p, which plays a protective role in intestinal IRI by inhibiting the TRAF3 signal pathway (Dai et al., 2019). The regulation of TRAF3 by non-coding RNA, mainly microRNAs (miRNAs), and long non-coding RNAs (lncRNAs), has also received attention in recent research of retinal IRI, which is common in diabetic retinopathy, glaucoma, and retinal vascular occlusive disorders, in which it leads to irreversible visual impairment and eventually results in blindness (Ge et al., 2020). In one study, methyl-CpG binding domain protein 2 (Mbd2), one of the DNA methylation readers, aggravated retinal cell apoptosis by targeting the Mbd2-associated long non-coding RNA 1 (Mbd2-AL1)/miR-188-3p/TRAF3 axis (Ge et al., 2020). However, the downstream pathways of TRAF3 were unexplored in retinal IRI. Therefore, based on the above research, it can be concluded that TRAF3 promotes injury induced by ischemia-reperfusion.

#### TRAF5

**FIGURE 3:**
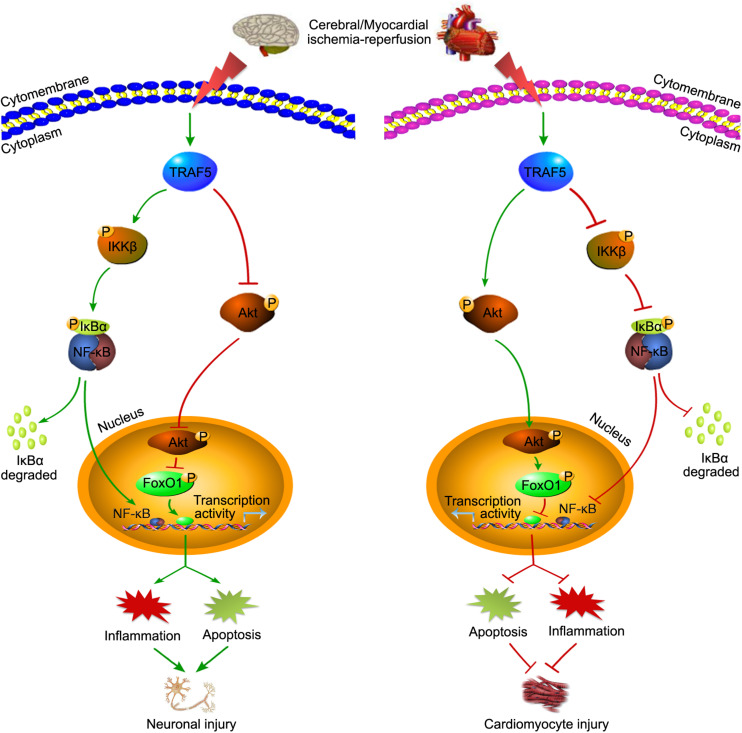
The contradictory roles of TRAF5 in cerebral and myocardial IRI. TRAF5 is activated with cerebral and myocardial ischemia-reperfusion. Activated TRAF5 procedurally phosphorylates and promotes the NF-κB pathway and inhibits the Akt/FoxO1 pathway, which promotes the transcription activity of FoxO1, leading to neuronal injury via promoting inflammation and apoptosis. The contradictory effects of TRAF5 are present in cardiomyocytes.

In addition to chronic inflammatory diseases, TRAF5 also plays important roles under various stresses, such as cerebral and myocardial IRI (Wang L. et al., 2013; Xu et al., 2020). However, based on the current research, TRAF5 seems to play a contradictory role in cerebral and myocardial IRI (Figure 3). In cerebral IRI, neuron-specific TG-TRAF5 mice exhibited aggravated blood brain barrier (BBB) disruption, more neuronal apoptosis, and increased inflammatory response compared with TRAF5-KO mice (Wang L. et al., 2013). Furthermore, cerebral IRI research proved that the deletion of TRAF5 inhibited NF-κB activity, but enhanced the Akt/FoxO1 pathway, in which FoxO1 is phosphorylated by p-Akt, leading to nuclear export and inhibiting transcription factor activity (Wang L. et al., 2013; Figure 3). Conversely, in myocardial IRI, TRAF5 deficiency both *in vivo* and *in vitro* exacerbated cardiomyocyte inflammation and apoptosis by activating the NF-κB pathway while inhibiting the Akt/FoxO1 pathway (Xu et al., 2020; Figure 3). The inconsistent effects of TRAF5 in cerebral and myocardial IRI are possibly due to the organ-specific function of TRAF5. Moreover, the expression of TRAF5 is low in various cell types of the heart, including fibroblasts, cardiomyocytes, and endothelial cells. Cardiomyocytes, along with other non-cardiomyocytes, possibly contribute to the synergistic effects of TRAF5 in myocardial IRI (Xu et al., 2020).

Additionally, the effects of TRAF5 on the NF-κB pathway are controversial. In agreement with the view about its role in myocardial IRI, TRAF5 deficiency aggravated cardiac dysfunction partly by activating NF-κB-dependent inflammatory responses under pressure overload, and the same view was verified in the research of inflamed colons (Bian et al., 2014; Shang et al., 2016). Meanwhile, there are studies suggesting that TRAF5 promoted the activation of the NF-κB pathway (Shang et al., 2016), which is more in line with the view of its role in cerebral IRI. Therefore, the effects of TRAF5 on IRI and the NF-κB pathway might be tissue- or disease-specific and need to be studied more thoroughly in the future.

#### TRAF6

**FIGURE 4:**
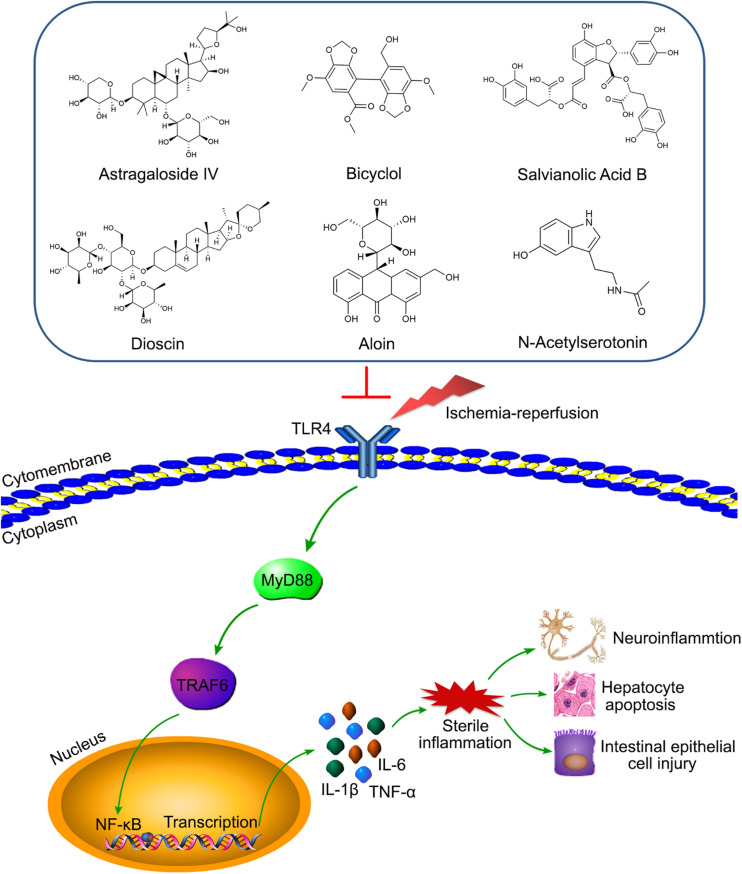
Various compounds target TLR4/MyD88/TRAF6/NF-κB axis to alleviate IRI of diverse organs. Astragaloside IV (AS-IV), bicyclol, salvianolic acid B (SAB), and dioscin relieve the neuro-inflammation by inhibiting the axis. Aloin and N-Acetylserotonin (NAS) reduce hepatocyte apoptosis and intestinal epithelial cell injury via targeting the axis, respectively.

As an intermediate mediator between receptors and pathways, TRAF6 is highly involved in IRI of various organs. For example, according to our knowledge, the TLR4/MyD88/TRAF6/NF-κB axis contributes to the injuries induced by ischemia-reperfusion of multiple organs, including the myocardia, liver, kidney, and retina (Li et al., 2010; Qi et al., 2014; Wang X. et al., 2014; Shao et al., 2016). Meaningfully, many drugs or compounds can reduce cerebral IRI targeting the axis, including Astragaloside IV (AS-IV), bicyclol, salvianolic acid B (SAB), and dioscin (Zhang et al., 2013; Tao et al., 2015; Wang et al., 2016; Li M. et al., 2017; Figure 4). Moreover, aloin and N-Acetylserotonin (NAS) were respectively involved in the regulating of liver and intestinal IRI by targeting TRAF6 (Du et al., 2019; Sukhotnik et al., 2019; Figure 4), which provides targets for clinical treatment of liver and intestinal IRI-related diseases. In addition, miRNA-based treatments for IRI are very popular in recent years (Huang Z. et al., 2019; Liang et al., 2019). Many studies have shown that the increased expression of miR-146a could inhibit TRAF6 to alleviate IRI of various organs, including the myocardia, kidney, intestine, and liver (Chen et al., 2013; Wang X. et al., 2013; Jiang et al., 2014; Dai et al., 2016; He et al., 2018). However, it is possible that TRAF6 was not significantly involved in the miR-146b/NF-κB pathway in regulating liver IRI (Zhang et al., 2017). For IRI treatments other than drugs, other treatment strategies are also extremely important, such as ischemic preconditioning (IPC) and ischemic postconditioning (IPostC). A study has shown that IPC could inhibit the TLR4/TRAF6 pathway to alleviate intestinal IRI (Liu S. Z. et al., 2017). Analogously, limb remote ischemic postconditioning (LRIP) played a protective role in cerebral IRI by inhibiting the MyD88/TRAF6/p38-MAPK pathway (Chen et al., 2016). Therefore, the above studies have demonstrated that targeting the TRAF6 pathway may provide a clear direction and excellent effect for IRI treatment.

However, there is a major challenge for the treatment of lung IRI, a common disorder in patients with lung transplantation, resuscitation for circulatory arrest, cardiopulmonary bypass, and pulmonary embolism, due to the fact that its molecular mechanisms may be more complicated than other organs (Liu X. et al., 2017; Shen et al., 2018). Moreover, the lungs seem especially vulnerable to IRI because of their dual blood supply systems and higher physiological demand for gas exchange and oxygen uptake (Liu X. et al., 2017). Fortunately, studies have shown that reducing the K63-linked ubiquitination of TRAF6 and inhibition of the downstream NF-κB and MAPK pathways could relieve the inflammatory response amplified by autophagy in lung IRI (Liu X. et al., 2017; Shen et al., 2018), suggesting that the ubiquitinated activation of TRAF6 and its management of autophagy are important underlying molecular mechanisms in lung IRI. Similarly, inhibition of the E3 ligase activity of TRAF6 significantly reduced cerebral and liver IRI (Li T. et al., 2017; Liu et al., 2020). Interestingly, TRAF6 could be inhibited by human mesenchymal stem cells (hMSCs)-derived exosomes, a kind of microvesicle with a diameter of about 30–100 nm, thereby reducing autophagy and apoptosis, playing a protective role in myocardial IRI (Jiang et al., 2018). Given all the above factors, we believe that the biological functions of TRAF6 and its related signaling pathways play a fatal role in the process of IRI.

## Conclusion

IRI is a common pathological process which occurs frequently in ischemic stroke, surgical treatment such as resection and transplant, thromboembolic events, and various other clinic events requiring the restoration of blood supply after ischemia, causing serious damage to various organs and even tissues throughout the body, thereby leading to a poor prognosis and high mortality. However, the pathogenesis of IRI is extremely complicated and varies in each organ, which brings enormous challenges to its treatment.

Explosive oxidative stress and inflammation response caused by various abnormal stimuli are common in the pathological process of IRI. Excessive accumulation of reactive oxygen species (ROS) and reactive nitrogen species are important pathological factors causing IRI in various organs, including retina, cerebral, myocardial, liver, kidney, lung and intestine (Jia et al., 2019, 2020; Li et al., 2020; Li et al., 2019d; Lonati et al., 2019; Qin et al., 2019; Yi et al., 2020). Oxidative stress may promote the expression of pro-inflammatory regulatory factors, and inflammatory cells may similarly induce the overproduction of ROS, thus forming a vicious circle to promote the occurrence and development of various diseases, including IRI. TRAFs are important mediators of inflammatory signaling and ROS regulation. For example, TRAF2 and TRAF6 are major components of ASK1 signalosome, which is very sensitive to oxidative stress and promote subsequent apoptosis (Noguchi et al., 2005; Sakauchi et al., 2017). Moreover, TRAF1 knockout effectively alleviate acute lung injury via inhibiting oxidative stress, inflammation and apoptosis (Bin et al., 2019). Further, as the adaptor proteins and the RING type E3 ubiquitin ligases, the TRAF family plays an indispensable role in the pathogenesis of IRI. The functions and activations of TRAFs are complex. Commonly, TRAFs are now recognized to be involved in a variety of signal cascades and act as central regulators of inflammation and immunity, including innate immune and adaptive immune. For instance, as the three main pattern recognition receptors (PRRs) of the innate immune system, TLRs, RLRs, and NLRs recruit TRAFs via MyD88 or TRIF, MAVS, and RIP2, respectively (Xie, 2013). TRAFs thereby regulates the downstream signaling pathways, including NF-κB, MAPK and interferon-regulatory factors (IRFs) pathways, to exert different biological effects, such as apoptosis, autophagy, necroptosis, and ferroptosis (Xie, 2013), which are fatal pathological processes in IRI.

Based on current research, we and other researchers have confirmed that most members of the TRAF family, except TRAF4 and TRAF7, play important roles in IRI by regulating different physiological mechanisms, including apoptosis, autophagy, necroptosis, and ferroptosis. Although TRAF proteins possess a similar structure, for IRI in diverse organs, each TRAF member exerts different effects by regulating different mechanisms, and even the same TRAF protein may cause different effects in different context. Visibly, TRAF2 knockdown increased the necroptosis of microglial and HT-cells under ischemic condition, but reduced neuronal apoptosis-induced by OGDR. Moreover, TRAF5 appeared to have a contradictory role in regulating cerebral and myocardial IRI. Therefore, TRAF proteins may exert different effects depending on the cell type and context-specific. Through gain and loss of function approaches, many studies have confirmed the definite molecular signaling pathways and effects of different TRAF members in regulating IRI of various organs, and the results have been summarized in Table 1, which showed that deletion or overexpression of TRAF molecules can cause obvious phenotypic differences in the models of various IRI, both *in vivo* and *in vitro*. In summary, the content presented in this review provides a compelling theoretical basis for IRI research and suggests clear targets for the treatment of IRI-related diseases.

**TABLE 1:** Genetic alterations of TRAF molecules in IRI or H/R models.

**TRAFs**	**IRI or H/R**	**Mice or cells genotype**	**Disease phenotype**	**Signaling pathways**	**References**
TRAF1	Cerebral IRI or H/R of primary neurons	Mice: neuron-specific TG-TRAF1; TRAF1-KO; Cells: Ad-TRAF1; Ad-shTRAF1	TRAF1 enlarged ischemic lesions and elevated neuronal apoptosis.	TRAF1/ASK1	Lu et al., 2013
	Liver IRI or H/R of primary hepatocyte or AML12 cells	Mice: hepatocyte-specific TG-TRAF1; TRAF1-KO; Cells: Ad-shTRAF1; Ad-TRAF1; pcDNA-TRAF1	TRAF1 aggravated liver histological injury, increased serum ALT/AST levels, and promoted cell apoptosis and inflammation.	TRAF1/ASK1/JNK; miR-214/TRAF1/ASK1/JNK	Zhang et al., 2014; Huang X. et al., 2019
	Myocardial IRI or H/R of primary cardiomyocytes	Mice: TRAF1-KO; Cells: Ad-TRAF1	TRAF1 aggravated the heart function, and promoted inflammation, and cardiomyocytes apoptosis.	TRAF1/ASK1/JNK/p38	Xu W. et al., 2019
TRAF2	Myocardial IRI	Mice: MHC-TRAF2_*L*__*C*_; MHC-TRAF2_*D*__*N*_	MHC-TRAF2_*L*__*C*_ mice had a lower LV developed pressure, a lower CK release, and a lower Evans blue dye.	TRAF2/NF-κB	Burchfield et al., 2010; Tzeng et al., 2014
	Intestinal IRI or H/R of Caco-2 cells	Cells: phospho-mutant TRAF2 plasmids: TRAF2-S55A (abolish phosphorylation); TRAF2-S55 (mimic phosphorylation)	TRAF2 Ser^55^ phosphorylation reduced cell apoptosis.	TRAF2/NF-κB/c-JUN	Zhou W. et al., 2017
	Cerebral IRI or H/R of primary microglia and neurons or HT-cells	Mice: TRAF2 shRNA lentivirus; Cells: TRAF2 shRNA lentivirus for HT-cells or TRAF2 siRNA plasmid for microglia	TRAF2 knockdown increased neuroinflammation, infarct volumes, and the necroptosis of microglial and HT-cells but reduced neuronal apoptosis.	TRAF2/MLKL; Sphk1/TRAF2/NF-κB	Su et al., 2017; Li et al., 2019b
TRAF3	Cerebral IRI or H/R of primary neurons	Mice: neuron-specific TG-TRAF1; neuron-specific TRAF1-KO; TRAF3^*flox/flox*^ mice; Cells: Ad-TRAF3; mutated TRAF3 (Ad-TRAF3-M); Ad-shTRAF3	TRAF3 aggravated neuronal loss, enlarged infarcts and promoted neuronal apoptosis.	TRAF3/TAK1	Gong et al., 2015
	Liver IRI or H/R of primary hepatocyte	Mice: hepatocyte-specific TRAF3 knockout (TRAF3-LKO); myeloid cell-specific TRAF3 knockout mice (LysM-TRAF3-KO); hepatocyte-specific TRAF3-TG (TRAF3-LTG); TRAF3^*flox/flox*^ mice; Ad-TRAF3; Ad-TRAF3-M; Cells: Ad-TRAF3; Ad-TRAF3-M	TRAF3 aggravated liver histological injury, increased serum ALT/AST levels, and promoted inflammation and cell death.	TRAF3/TAK1/JNK/NF-κB	Hu et al., 2016
	Myocardial IRI or H/R of primary cardiomyocytes	Mice: TRAF3 siRNA *in vivo* transfection; Cells: TRAF3 siRNA	TRAF3 knockdown reduced the infarction, attenuated cardiac histological, decreased CK-MB release, and alleviated cell apoptosis, inflammation and oxidative stress.	TRAF3/JNK	Liu X. et al., 2018
	Intestinal IRI or H/R of IEC-6 cells	Cells: TRAF3 overexpression and siRNA plasmid	TRAF3 promoted inflammation and cell apoptosis.	miR-29b-3p/TRAF3	Dai et al., 2019
TRAF5	Cerebral IRI	Mice: neuron-specific TG-TRAF5; TRAF5-KO	TRAF5 aggravated BBB disruption, augmented infract volumes, increased neuronal apoptosis inflammatory response.	TRAF5/Akt/FoxO1	Wang L. et al., 2013
	Myocardial IRI or H/R of primary cardiomyocytes	Mice:TRAF5-KO Cells:Ad-TRAF5	TRAF5 relieved infract size, improved cardiac dysfunction, reduced cardiomyocytes apoptosis inflammatory response.	TRAF5/Akt	Xu et al., 2020
TRAF6	Liver IRI or H/R of RAW264.7 cells	Cells: TRAF6 siRNA	TRAF6 silencing reduced proinflammatory cytokine production.	miR-146a/TRAF6/IRAK1	Wang X. et al., 2013
	Cerebral IRI or H/R of primary neurons	Mice: neuron-specific TG-TRAF6; neuron-specific TRAF6-KO; Cells: Ad-TRAF6; Ad-TRAF6-M; Ad-shTRAF6	TRAF6 increased infract volumes, neurological deficit scores, promoted inflammation, oxidative stress and cell apoptosis.	TRAF6/Rac1	Li T. et al., 2017
	H/R of IEC-6 cells	Cells: Ad-TRAF6	Overexpression of TRAF6 promoted cell apoptosis.	miR-146a/TLR4/TRAF6/NF-κB	He et al., 2018
	Myocardial IRI or H/R of HCM cells	Cells: pcDNA3.1-TRAF6	Overexpression of TRAF6 promoted inflammatory response cell apoptosis.	lncRNA ROR/miR-124-3p/TRAF6	Liang et al., 2019

## Perspectives

IRI is extremely common in clinical treatment and can cause serious consequences. Although many studies have shown that some treatments, such as IPC and IPostC, could improve IRI, there is still a lack of precise treatment strategies in the clinical setting currently. The points presented in this review indicated that TRAFs have an important influence in the development of IRI. Manipulation of TRAF proteins or the molecular pathways they regulate can provide new ideas for the treatment of IRI-related diseases. In fact, studies have shown that IPC and LRIP could significantly improve intestinal and cerebral IRI by targeting TRAF6 pathways (Chen et al., 2016; Liu S. Z. et al., 2017). In addition, a recent study showed preactivated and disaggregated shape-changed platelet (PreD-SCP) therapy effectively reduced the renal IRI by inhibiting the TLR4/MyD88/TRAF6 signaling pathway (Chen et al., 2019). In addition to the research on treatment methods, small agonists and antagonists targeting TRAFs have also became a focus in recent years. Numerous studies have shown that different miRNAs could reverse the effects of targeting respective TRAFs and reduce the IRI of various organs, including the liver, heart, intestine, kidney, and retina (Dai et al., 2016, 2019; Huang Z. et al., 2019; Liang et al., 2019; Ge et al., 2020). Moreover, many drugs or compounds played a protective role in IRI by targeting the TRAFs pathways. Illustratively, tauroursodeoxycholic acid (TUDCA), a classical conjugated bile acid, can effectively alleviate liver IRI via inhibiting IRE1α/TRAF2/NF-κB pathway activity (Xu et al., 2018). Furthermore, AS-IV, bicyclol, SAB, and dioscin all played protective roles in cerebral IRI by inhibiting the TLR4/MyD88/TRAF6/NF-κB axis (Zhang et al., 2013; Tao et al., 2015; Wang et al., 2016; Li M. et al., 2017; Figure 4). Additionally, the latest research showed that TRAF6 was also involved in the regulation of aloin and NAS preconditioning to reduce liver and intestinal IRI, respectively (Du et al., 2019; Sukhotnik et al., 2019; Figure 4). The above research indicated that TRAFs have received much attention in the treatment of IRI, and new treatment methods and related mechanisms have been continuously discovered. However, the targets of TRAF proteins in various organs and the signaling pathways they regulate are variational; even TRAF proteins may have opposite effects in different organs, which brings great obstacles to the research of drugs or strategies for the treatment to IRI-related diseases. Moreover, it is still unclear whether TRAF4 or TRAF7 could also play an important role in IRI like other TRAF members, and what kind of specific role they would play. Therefore, the role of TRAFs in IRI needs more in-depth studies in the future.

## Author Contributions

WZ and QY formulated the conception of this review. WZ and DL co-wrote the manuscript. ZZ and QY revised the manuscript. All authors read and approved the final manuscript.

## Conflict of Interest

The authors declare that the research was conducted in the absence of any commercial or financial relationships that could be construed as a potential conflict of interest.

## Abbreviations

ALDOA: aldolase A
aPKC: atypical protein kinase C
ASK1: apoptosis signal-regulating kinase 1
AS-IV: Astragaloside IV
BBB: blood brain barrier
BMAL1: aryl hydrocarbon receptor nuclear translocator-like protein 1
CK: creatine kinase
CSFV: classical swine fever virus
EAD: early allograft dysfunction
ER: endoplasmic reticulum
EV71: enterovirus 71
HBV: hepatitis B virus
HCC: hepatocellular carcinoma
hMSCs: human mesenchymal stem cells
HPV: human papilloma viruses
H/R: hypoxia/reoxygenation
IBD: inflammatory bowel disease
IFN-I: type I interferon
IPC: ischemic preconditioning
IPostC: ischemic postconditioning
IRI: ischemia-reperfusion injury
lncRNA: long non-coding RNA
LPS: lipopolysaccharide
LRIP: limb remote ischemic postconditioning
LV: left ventricular
MAPK: mitogen-activated protein kinase
Mbd2: methyl-CpG binding domain protein 2
miRNAs: microRNA
MLKL: mixed-lineage kinase domain-like
NAS: N-Acetylserotonin
NF- κ B: nuclear factor- κ B
NLR: NOD-like receptor
NLS: nuclear localization signal
OGDR: oxygen-glucose deprivation reperfusion
ORF3a: open reading frame 3a
PI3K: phosphatidylinositol-3-kinase
PreD-SCP: preactivated and disaggregated shape-changed platelet
RLR: RIG-I-like receptor
SAB: salvianolic acid B
SARS-CoV: severe acute respiratory syndrome coronavirus
SLE: systemic lupus erythematosus
Sphk1: sphingosine kinase 1
TAK1: transforming growth factor- β -activated kinase 1
TGF-β: transforming growth factor-β
TLR: Toll-like receptor
TRAF: Tumor necrosis factor receptor-associated factor
TUDCA: tauroursodeoxycholic acid.
